# First description of injury feigning behavior in Godlewski's buntings

**DOI:** 10.1002/ece3.10028

**Published:** 2023-04-18

**Authors:** Xizhu Liu, Yuhan Zhang, Guixia Wan, Haixia Luo, Longwu Wang, Wei Liang

**Affiliations:** ^1^ School of Life Sciences Guizhou Normal University Guiyang China; ^2^ Ministry of Education Key Laboratory for Ecology of Tropical Islands Key Laboratory of Tropical Animal and Plant Ecology of Hainan Province College of Life Sciences Hainan Normal University Haikou China

**Keywords:** Godlewski's bunting, ground‐nesting birds, injury feigning behavior, nest defense, nest predation

## Abstract

Nest defense behavior helps ground‐nesting birds improve their breeding success. Among such behaviors, injury feigning behavior (IFB) is one of the better ways to attract predators and protect birds' nests. IFB is generally associated with shorebirds in general and plovers in particular, however, through field observation, it has been found this behavior is also exhibited in bunting species when they facing the risk of predation. We attempt to explore the detailed description of this behavior in buntings and the factors that affect this behavior. Based on video‐recordings of the nest defense behavior by the ground‐nesting bird Godlewski's bunting (*Emberiza godlewskii*) against nest invaders, we explored the characteristic of IFB and differences in nest defense behavior during their brooding seasons. The results showed that female buntings had a distinct IFB: the wings of buntings incited within 60°–90° of the body, ran and called rapidly, pretending to be injured and unable to fly. The nest stage had significant and extremely significant effects on IFB and movement distance (MD), respectively. And IFB was more likely to occur in brooding (34.62%) than incubation period (16.42%). This indicates that there are significant differences in the IFB of buntings at different breeding stages.

## Graphical abstract

Based on video‐recordings of the nest defense behavior by the ground‐nesting bird Godlewskiʼs bunting (*Emberiza godlewskii*) against nest invaders, we explored the characteristic of IFB and differences in nest defense behavior during their brooding seasons. The results showed that female buntings had a distinct IFB: the wings of buntings incited within 60°–90° of the body, ran and called rapidly, pretending to be injured and unable to fly.

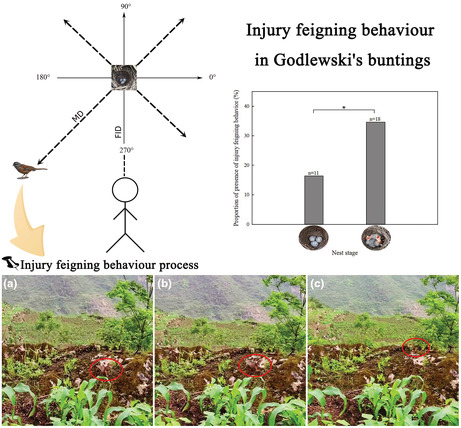

## 1 INTRODUCTION

Nest predation is one of the main causes of reproductive failure in birds. Approximately 80% of failures are caused by nest predation, especially in small passerine birds that build open nests (Martin, 1992; Martin & Roper, 1988; Sanders & Maloney, 2002). Nest predation is an important selective pressure in the formation of bird behavior, thereby prompting parents to evolve appropriate antipredator strategies to cope with nest predation to maximize their fitness (Ghalambor & Martin, 2001; Hua et al., 2014; Martin et al., 2000). Among them, nest defense is one of the effective antipredator strategies for birds (Clutton‐Brock, 1991; Smith & Edwards, 2018), which reduces the nest predation risk and improves offspring survival (Krams et al., 2014; Montgomerie & Weatherhead, 1988; Zub et al., 2017).

Because parents face the risk of predation during nest defense, they make trade‐offs between their own and offspring survival (Ghalambor & Martin, 2001). In the presence of a potential predator situation, parents optimize their own defense behavior to ensure maximum gain (Brunton, 1986; Humphreys & Ruxton, 2020; Murphy, 1926). When they encounter potential predators, some parents place themselves at the most immediate risk to ensure offspring survival, diverting the attention of predators from their offspring to themselves to protect their offspring (Caro, 2005; De Framond et al., 2022; Gochfeld, 1984; Humphreys & Ruxton, 2020). This behavior is called injury feigning behavior (hereafter IFB), which is the way some birds use to protect eggs or nestlings in nests (Barash, 1975; Davis Jr, 1989; Kameda, 1994; Tseng et al., 2017). Including the Alpine Accentor (*Prunella collaris*) (Barash, 1975), Pauraque (*Nyctidromus albicollis*) (Aragonés, 1997), Savanna Nightjar (*Caprimulgus affinis*) (Tseng et al., 2017), and Golden‐crowned Warbler (*Basileuterus culicivorus*) (Smith, 2017), IFB is among their nest defense behaviors when faced with predators.

Although parents' IFB can increase the survival of offspring, they face losses such as being preyed upon or consuming energy (Brunton, 1986, 1990; Byrkjedal, 1987; Frid & Dill, 2002). Therefore, parents adjust the frequency of IFB during the breeding season to achieve a balance between reproductive input and their own survival (Tseng et al., 2017). In birds, it is typical to use IFB to protect offspring, especially in Charadriiformes birds (Humphreys & Ruxton, 2020). Parents may tend to protect older offspring because older offspring bring greater benefits (Regelmann & Curio, 1983). For example, Halupka (1999), in his study of Aquatic Warblers (*Acrocephalus paludicola*), found that parents invest more as their offspring get older, with offspring age significantly associated with female response.

For ground‐nesting birds, which are at high risk of predators, nest defense behavior plays an important role in breeding success (Gómez‐Serrano & López‐López, 2016). In the study of breeding ecology of the Godlewski's bunting (*Emberiza godlewskii*) (hereafter bunting), we found that it exhibited IFB when it faced predation risk in both the incubation period and brooding period. Through field experiments and observations, we recorded nest defense behavior in bunting facing potential predators during the incubation period and nestling period. We recorded the detailed display process of buntings IFB, and explore the factors that influence the nest defense behavior of bunting by relating bunting's behavior to clutch or brood size and nest environmental factors.

## 2 MATERIALS AND METHODS

### 2.1 Study areas and species

The study site is located in Liuzhi (26°10–26°14′ N, 105°13′–105°24′ E), Guizhou, southwestern China, with an altitude of 1,287–1,657 m, which is a subtropical monsoon climate area. The study area features a karst mountainous landscape, consisting of villages, arable lands, rivers, shrubs, and barren slopes.

Bunting is a small songbird of the genus Emberiza, family Emberizidae in the order Passeriformes. Its clutch size is usually 2–4, and it is mainly hatched by female buntings. They belong to altricial birds and are widely distributed in China (Zhao, 2001). They are typically ground‐nesting birds, nesting in farmlands or mountains.

### 2.2 General field methods

We systematically searched bunting habitats during the bird breeding season of 2021 and 2022 (April–August). We numbered the new nests, used GPS to position them and recorded the habitat type (cropland, barren mountain, and abandoned farmland) and nest sites (under the grass roots, under the lacclith and soil pit). Measured the nest height that distance from nest to horizontal ground by using an infrared range finder (DELIXI ELECTRIC, DE series, Range: 0–50 m).

**FIGURE 1:**
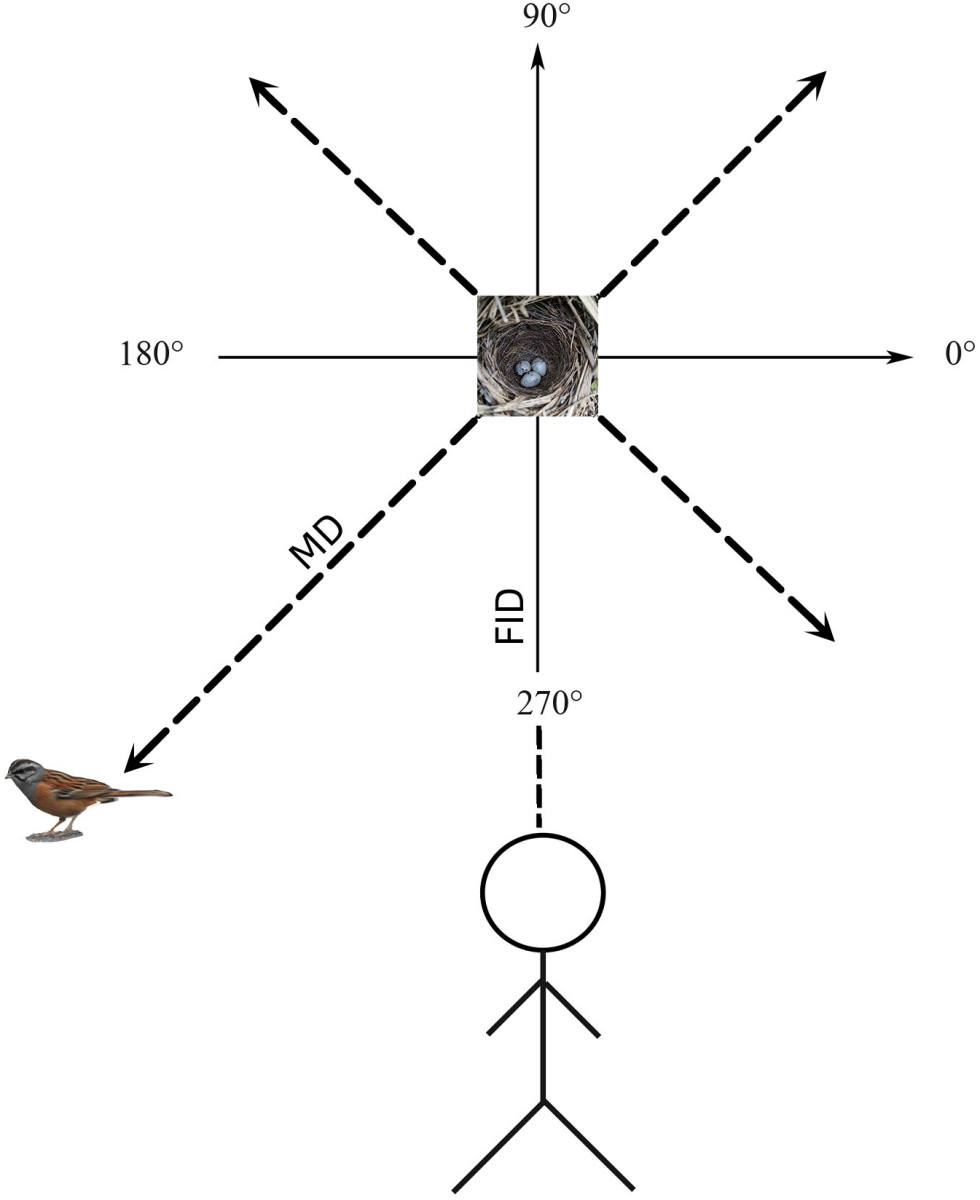
A diagram of the simulation experiment of the observer simulating the human (the potential predator) approaching the Godlewski's bunting nests in the field. Coordinate axis and dotted line: the direction in which the female Godlewski's bunting flies out of the nest; FID, flight initiation distance; MD, movement distance.

Field experiments on bunting nest defense behavior were performed in the incubation and brooding period. The nest in the incubation period may continue to do experiments in the brooding period after hatching, but with an interval of at least 3 days. All experiments were performed under good weather conditions and the experimental time was from 7:00 a.m. to 7:00 p.m. Choosing the time when the female bunting was incubating eggs or hatching chicks, the researcher simulated that potential predators were approaching the nest at a constant speed (0.5 m/s) on the front of the nest until the female bunting left the nest. The distance between the nest and the person when the bird left the nest called the flight initiation distance (hereafter “FID”), and the distance from the first landing position to the nest after the female bunting flies out of the nest is the movement distance (MD). We recorded the direction toward, which the female bunting flies out and whether the female bunting moves back after it stops flying and measured the FID and MD using an infrared range finder. The experimental simulation diagram was shown in Figure 1. We also recorded the process with a camera or mobile phone and measured the distance it is presented if the bunting exhibits IFB.

Each experimental nest was selected for observation and recording after full clutch size and nestlings that are 1–3 days of age. Because female buntings were present in the nest for incubating eggs or keeping nestlings warm at this stage to facilitate the experiment. The experiment was performed by the same researcher who wore the same clothing.

### 2.3 Data analysis

The factors potentially associated with the IFB and MD were evaluated using generalized linear mixed effects models (GLMMs). The selected fixed effects are clutch or brood size (1–4; numerical variable), nest stage (incubation period; brooding period; categorical variable), habitat type (cropland, barren mountain, abandoned farmland; categorical variable), nest height (numerical variable), and nest sites (under the grass roots, under the laccolith, soil pit; categorical variable). Nest ID was included as random effects to account for variation in the intrinsic frequency (random intercept) of IFBs across nests.

Statistical analyses were performed using IBM SPSS 26.0 (IBM Inc.). Values are presented as the mean ± SD unless stated otherwise.

## 3 RESULTS

### 3.1 Description of injury feigning behavior

We found 114 bunting nests in the wild and performed the researcher's simulated invasion response experiment on 67 valid experimental nests, in which 11 nests in the incubation period and 18 in the brooding period were recorded to have IFB.

**FIGURE 2:**
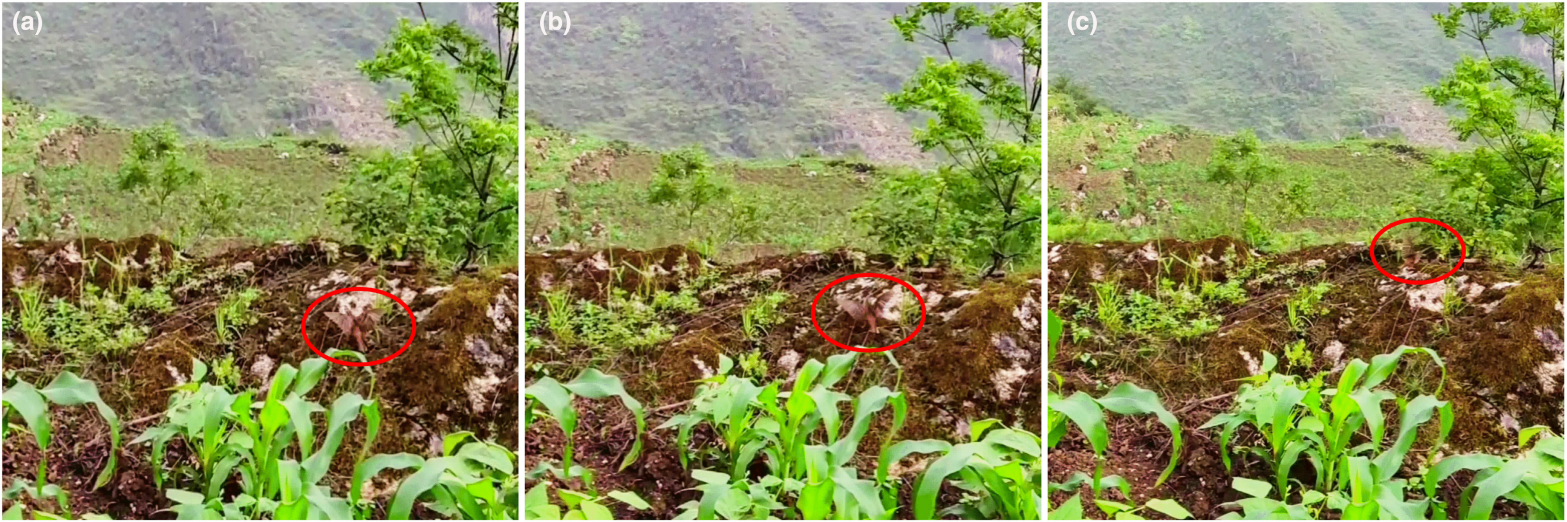
Injury feigning behavior process of Godlewski's bunting in the field. (a–c) The red circle shows the female Godlewski's bunting.

The typical IFB process was as follows: when the researcher approached the bunting nest for a certain distance (FID, 1.035 ± 0.633 m, *n* = 29), 79.31% (23/29) of the female buntings would fly out of the nest in the direction of 180°–360° and stop not far from the nest (MD, 2.580 ± 1.903 m, *n* = 29). Most of the female buntings would look back (93.10% 27/29) and lift their wings gradually away from the nest to run away from the nest, and the wings incited within 60°–90° of the body (Figure 2), like shaking their wings after injury when they could not fly up, accompanied by rapid calling. Then they would lift their wings to fly some distance (2.008 ± 1.093 m, *n* = 29) and then fly to nearby shrubs or grass and return to their nests after the researcher left for a time period (See Video S1).

### 3.2 The affecting factors of nest defense behavior

**FIGURE 3:**
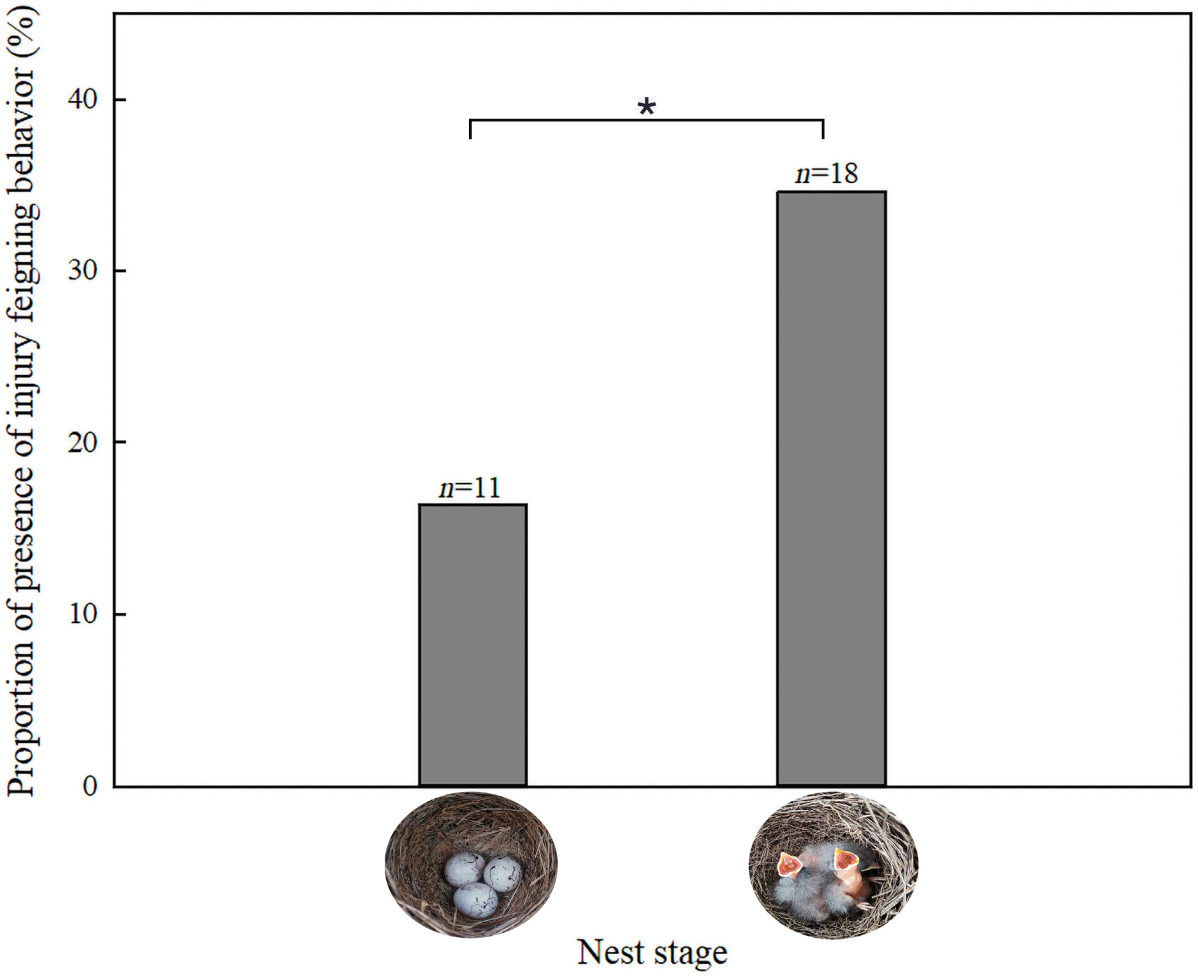
Proportion of IFB displayed by Godlewski's bunting at different stages. The figure represents the proportion of IFB in buntings in the incubation period and brooding period.

According to GLMMs analysis, the frequency of IFB in bunting was only significantly affected by nest stage (*F*_1,109_ = 4.690, *p* = .033), but not by other nest‐related factors such as environmental factors (see Table. 1). The random effect nest ID (*Z* = 0.629, *p* = .530 > .05) intercept variance was 0.492. We further analyzed the nest stage and found that female bunting' IFB in the incubation period had a probability of 16.42% (11/67) and a probability of 34.62% (18/52) in the brooding period, and the probability of the brooding period was more than twice that of the incubation period. Moreover, the brooding period showed more significant differences in IFB than the incubation period (Chi‐squared test, *χ^2;^ 1* = 7.496, *p* = .022) (Figure 3). Among the three different nest sites and habitats, the probability of IFB increased in the brooding period, except in nest sites in pit soil.

**TABLE 1:** GLMM analyses of the experiment to test whether IFB refers to clutch or brood size, nest stage, habitat sites, nest height, and nest sites of Godlewski's bunting nests.

Source	*F*	*df*	*df*	*p*
Clutch or brood size	0.752	3	109	.523
Nest stage	4.690	1	109	.033*
Habitat type	0.579	2	109	.562
Nest height	0.489	1	109	.486
Nest sites	0.443	2	109	.643

* *p* < .05.

**FIGURE 4:**
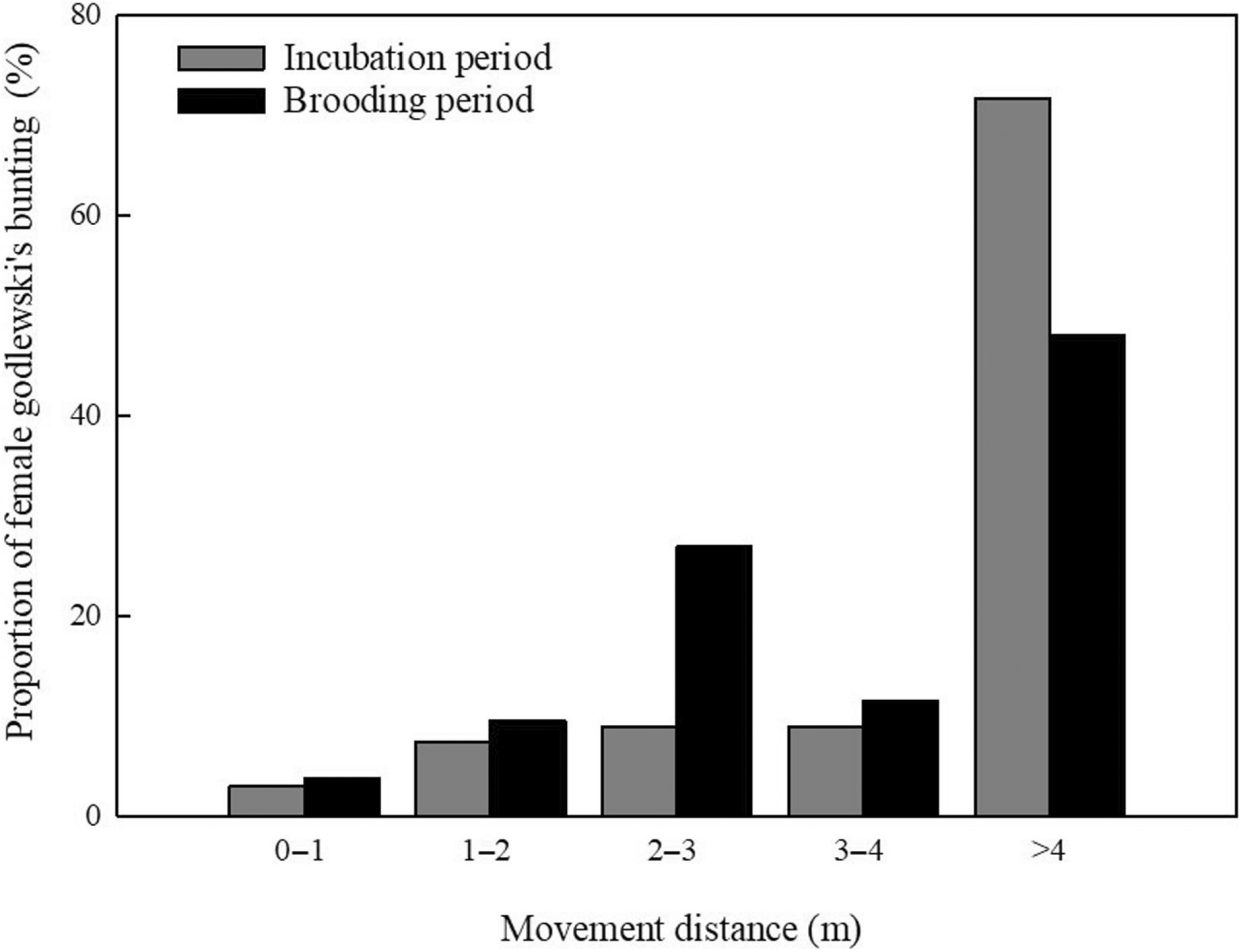
Proportion of MD of female Godlewski's buntings at different stages.

In incubation period and brooding period, the MD and FID of the bunting were also different. According to GLMMs analysis, the frequency of MD in bunting was only extremely significantly affected by nest stage (*F*_1,109_ = 7.185, *p* = .008), but not by other nest‐related factors such as environmental factor (see Table 2). The variance of the random effect nest ID intercept is 8.335, which is statistically significant (*Z* = 3.691, *p* = .0002 < .01), indicating that the intercept is random. The probability of the incubation period having a MD of greater than 4 m was 71.64% (48/67), which was much higher than that of the brooding period (48.08%, 25/52) (Figure 4.), and the values showed a highly significant difference (Chi‐squared test, *χ^2^ 1* = 7.496, *p* = .002). The FID of brooding period female buntings ranged from 0 to 1 m in 42.31% (22/52), while the incubation period accounted for a slightly lower proportion than the brooding period (38.81%, 26/67).

**TABLE 2:** GLMMs analyses of the experiment to test MD, clutch or brood size, nest stage, habitat sites, nest height and nest sites of Godlewski's bunting nests.

Source	*F*	*df*	*df*	*p*
Clutch or brood size	0.435	3	109	.728
Nest stage	7.185	1	109	.008**
Habitat type	1.033	2	109	.359
Nest height	0.231	1	109	.632
Nest sites	0.288	2	109	.750

** *p* < .01.

## 4 DISCUSSION

The video recording showed that the actual IFB process of female buntings in the field. It is the first time that the IFB has been recorded in buntings, and a detailed description of the behavior has been provided. The results show that the nest stage has significant influence on IFB, and the frequency of IFB in brooding is higher than that in incubation, which indicates that IFB may be one of the important nest defense behavior of buntings. Changes in MD and FID also indicate buntings nest defense behavior is enhanced in brooding period. Previous studies on avian IFB, especially those of Charadriiformes, are quite common (Bengtson, 1970; Brunton, 1990; Byrkjedal, 1991; Graul, 1971; Humphreys & Ruxton, 2020). In the order Caprimulgiformes Caprimulgidae (Aragonés, 1997; Steyn, 1971; Tomkins, 1942; Tseng et al., 2017), Passeriformes Parulidae and Parulidae (Barash, 1975; Smith, 2017; Taverner et al., 1936) and others have also been reported. But buntings have not been reported of IFB in the studies that have been done so far. The IFB description of the Alpine Accentor (*Prunella collaris*) and Purple Sandpiper (*Calidris maritima*) is that they stretch out and shiver their wings like an injury, and run with a high‐pitched chirp. The behavior of these two birds is similar to the IFB of buntings in this study.

Nest defense behavior is the most direct way to avoid nest predation, but it also consumes a lot of energy from parents and increases predation risk (Kameda, 1994). Thus, in most cases, parents weigh their own risks as well as offspring inputs (Coleman et al., 1985; Trivers, 1972). As investment of parents for offspring increases, sometimes parents will make themselves bear greater risks to ensure the survival of the offspring (Montgomerie & Weatherhead, 1988), such as parents' nest defense behavior, which will greatly reduce the risk of predation of the offspring (Greig‐Smith, 1980; Redondo & Carranza, 1989; Weatherhead, 1990). Studies have shown that American golden plovers (*Pluvialis dominica*) with IFB have a higher nest survival rate than exposed birds (Byrkjedal, 1991). In addition, it has been concluded that the strength of nest defense increases as the breeding cycle progresses in Aquatic warblers, and female birds are more prone to risk of predator defense behavior in the brooding period than in the incubation period, such as a close proximity to potential predators (Halupka, 1999). It indicated that buntings tended to stop near the nest after fleeing out of the nest in the brooding period and would bear a greater risk to itself. FID can reflect risk trade‐offs for prey individuals during predator proximity (Blumstein, 2006; Møller, 2008), and longer FIDs imply lower probability of being preyed (Møller, 2014).

Compared with hatching, parents invest more in fledgling offspring and have a greater chance of successful nest reproduction, which will have greater value (Andersson et al., 1980), and parents' nest defense behavior will be stronger. In this study, when the bunting nest faced potential predation risk, IFB and MD has significant difference in different period, FID also becomes shorter in brooding period. The closer the distance is, the higher the predation risk its female buntings themselves may face. The change in bunting nest defense behavior suggests that as the nest age increases, its intensity increases, leading the bird to bear greater risks and invest more in improving offspring.

In conclusion, our research is the first description that the IFB is one of nest defense behavior in buntings, and the behavior was recorded in detail recorded. But, the specific investment changes of bunting in the brooding period may need more detailed data to discuss. The study on IFB of buntings may provide valuable insights into the evolution of the distraction behavior of avian species.

## AUTHOR CONTRIBUTIONS

**Xizhu Liu:** Data curation (equal); investigation (equal); writing – original draft (equal). **Yuhan Zhang:** Data curation (equal); investigation (equal). **Guixia Wan:** Investigation (equal). **Haixia Luo:** Investigation (equal). **Longwu Wang:** Supervision (equal); writing – review and editing (equal). **Wei Liang:** Supervision (equal); writing – review and editing (equal).

## FUNDING INFORMATION

This work was supported by the National Natural Science Foundation of China (Nos. 31960105, 32260253 to LW, 31970427, 32270526 to WL). LW was funded by the Guizhou Natural Science Foundation (No. ZK [2022]‐316), and WL supported by the specific research fund of The Innovation Platform for Academicians of Hainan Province.

## CONFLICT OF INTEREST STATEMENT

The authors declare that they have no competing interests.

## Supporting information


Video S1

## Data Availability

Data and video of all analyses presented in this manuscript were submitted as Video S1 at the Dryad Digital Repository: https://datadryad.org/stash/share/f8yi8I8Y‐gfnLMm66QFsu5WQnSlSGJKUrPNAIJbdnEY.
